# Community-Engaged Codesign and Piloting of the FOOD4MOMS Produce Prescription Program for Pregnant Latina Women

**DOI:** 10.1016/j.cdnut.2025.104572

**Published:** 2025-02-19

**Authors:** Sofia Segura-Pérez, Andrea Tristán Urrutia, Anqi He, Amber Hromi-Fiedler, Katina Gionteris, Kathleen O. Duffany, Elizabeth C Rhodes, Rafael Pérez-Escamilla

**Affiliations:** 1 Hispanic Health Council, Hartford CT, USA; 2 Department of Social and Behavioral Sciences, Yale School of Public Health, New Haven CT, USA; 3 Wholesome Wave, Bridgeport CT, USA; 4 Yale-Griffin CDC Prevention Research Center (PRC), New Haven and Derby, CT, USA; 5 Hubert Department of Public Health, Rollins School of Public Health, Emory University, Atlanta GA, USA

**Keywords:** food as medicine, produce prescription, pregnant women, Hispanic, community engaged, program design

## Abstract

**Background:**

Eating plenty of fruit and vegetables is key for maternal-child food and nutrition security. In the United States, fruit and vegetable consumption is lower among low-income families. Produce prescription programs (PRx) provide monetary benefits to low-income individuals to buy fresh produce or directly provide the produce itself to improve their food and nutrition security.

**Objective:**

To codesign the FOOD4MOMS PRx (F4M) program for low-income Hispanic pregnant mothers and to test its feasibility through a pilot study using the Program Impact Pathway framework.

**Methods:**

The participants for the codesign phase and the feasibility phase were recruited from the Hispanic Health Council Maternal Health Programs and the Special Supplemental Nutrition Program for Women, Infants and Children program. Listening sessions (LSs) with adult Hispanic pregnant and nonpregnant women with children aged <3 y were conducted and transcribed for thematic analysis to inform the F4M codesign. The pilot feasibility study only included adult pregnant women enrolled during the first 2 pregnancy trimesters.

**Results:**

The 3 codesign LSs (n = 21 participants) showed that participants thought good nutrition was very important during pregnancy and were very interested in having skill-building nutrition classes as part of the PRx. Most participants preferred receiving incentives through the Fresh Connect card to allow them to choose their produce. They also recognized that some participants with limited transportation options may benefit from a produce box delivered at home. All pilot study participants chose the electronic benefit transfer card as their incentive redemption channel. The redemption rate of benefits by pilot participants was 70% and they felt that F4M helped them and their families consume more produce. Pilot participants reported high levels of satisfaction with F4M.

**Conclusion:**

The community-engaged codesign approach likely explains the successful piloting of the feasibility of F4M and the strong satisfaction of the clients participating in it.

## Introduction

Produce prescription programs (PRx), part of the United States Food is Medicine strategy [1,2], provide incentives to people with low incomes to purchase fresh produce or receive produce through home delivery to improve their health and wellbeing [2,3]. PRx programs have predominantly focused on individuals at risk of or with chronic diseases [4]. These programs have been associated with reductions in household food insecurity, as well as improvements in fruit and vegetable intake, overall dietary quality, glucose control, hypertension, body weight, disease self-management, and self-perceived physical and mental health [[4], [5], [6], [7]]. Modeling studies show that PRx are cost effective [[4], [5], [6], [7], [8]]. Although some PRx have focused on early childhood or families with children [9], very few have focused on the first 1000 days of life, defined as gestation through the first 2 y after birth. Optimal nutrition during this period is crucial for maternal health and the healthy growth and development of their offspring [[10], [11], [12], [13], [14], [15], [16]]. Fresh fruits and vegetables are an excellent source of vitamins, minerals, and dietary fiber [14]. However, women with low incomes do not consume enough produce during pregnancy [[17], [18], [19], [20]]. A study conducted with low-income Hispanic pregnant women in New York using the Healthy Eating Index to assess dietary quality found that their overall dietary quality was suboptimal with a lower intake of vegetables (65.3%) and fruits (45%) than that recommended by Healthy Eating Index-2015 [21]; thus, there is a need to design and implement effective and person-centered PRx for pregnant and postpartum women and their young children [22].

Community-engaged codesign of an equitable PRx for pregnant women has strong potential to optimize the usability, person-centeredness, and delivery of PRx, and thereby maximize engagement and impact among clients [23]. Implementation science frameworks, particularly when integrated with behavior change theories like COM-B (Capability-Opportunity- Motivation-Behavior) [24], emphasize the need for programs to be codesigned, involving all essential interest-holders, while justifying why the activities involved with a program have the potential to impact the healthy lifestyle behaviors of interest. This includes potential program clients, program implementors, staff from community-based organizations, funders, and program evaluators [25].

The objectives of this study were to *1*) describe the codesign process followed for F4M and *2*) assess the feasibility and client satisfaction of the codesigned F4M through a pilot study.

## Methods

This study was conducted in 2 phases, a codesign phase that included sequential LSs to receive the input from mothers from the community on how to specifically implement each activity of the PRx, following a Program Impact Pathways (PIP) approach [26]. This phase also included mapping the PIP to the COM-B framework [24]. The second phase was designed to pilot the program based on the consensus final PIP to test its feasibility and clients’ program satisfaction. The specific details of how each phase was conducted are presented below.

### Study site

The City of Hartford, CT has a population of 119,669 habitants of whom 44.8% are Hispanic or Latinos. Among those who are Hispanic, 74% are Puerto Rican [27] 36.1% Black, and 15.9% non-Hispanic White, and 18% are children aged <18 y [28]. In Hartford, the median household income is $45,300, which is much lower than the state median household of $93,760. About 25.5% of Hartford live in poverty, which is more than double the overall poverty rate in the state (10.3%) [28]. Nearly two-thirds of Hispanic/Latina female-headed households with children <18 y (58%) live below poverty, followed by Puerto Ricans (54%) and the overall population (44%) of Hartford [28,29]. In 2023, 32% of the households in Hartford were food insecure, 54% reported being just getting by or struggling financially, and 37% of children were living in poverty [30]. The City of Hartford in CT is considered a food swamp, an area in which unhealthy food options are more prevalent than healthy ones [31], which is of concern because food stamps are a significant predictor of poor dietary quality and obesity [32]. Hence, Hartford is a city that could strongly benefit from F4M.

### Frameworks

This project utilized the PIP program implementation and monitoring approach [26] combined with the COM-B framework [24].

### Codesign phase

#### Listening sessions

We conducted 3 sequential LSs with Hispanic adult women living in the Hartford, CT area who were either pregnant (57%) or had a child aged <2 y (43%) to elicit their input on the design of F4M. Each LS included 4 to 9 women, with a total of 21 women participating in the sessions. Women were recruited through the Hispanic Health Council (HHC) maternal and child health programs and from the Special Supplemental Nutrition Program for Women, Infants and Children (WIC) offices by well-trained and supervised HHC community health workers with extensive experience in community-engaged research. Each LS lasted between 68 to 80 min; the first (*n* = 8) and third sessions (*n* = 4) were conducted in Spanish, and the second session (*n* = 9) was conducted in both English and Spanish. Before the session started, the study was explained to the participants, consent was obtained, and they were administered a brief sociodemographic survey. The LSs were guided by a semistructured questions list that probed for women’s knowledge and perspectives on nutrition and access, preparation, and consumption of fruits and vegetables during pregnancy, previous experiences with similar food assistance programs, and views and opinions on the design of each program step, including recruitment methods, incentives options (i.e., electronic benefit transfer [EBT] card, voucher, online ordering, and produce box delivery) (Table 1), and nutrition education (Supplementary Material - Table 1). These program steps were presented, along with feedback from previous sessions, at the beginning of each LS to elicit additional feedback. By the last session, no additional feedback on the main F4M program activities arose, and final decisions on incentives options were made considering preferences and feasibility. All LSs were moderated by the senior author (RP-E), and SS-P took notes throughout the sessions conducted at HHC.

**TABLE 1:** FOOD4MOMS incentive options discussed with Hispanic women attending the program codesign listening sessions in Hartford, CT.

Proposed incentive options	Description
Fresh Connect EBT card	Participants would receive a Fresh Connect EBT card and be credited with $100 USD at the start of each month for 10 mo. The EBT card could be used at specific grocery or food retail stores. Any unused balance on the card would not be carried over to the subsequent month.
Voucher^1^	Participants would be provided with vouchers for redemption at grocery stores.
Online ordering^1^	Participants would order fruits and vegetables online from a grocery store’s website or application, pay with an EBT card, and then the produce would be delivered to their homes by the store.
Produce box^1^	The produce boxes would be delivered to the participants' homes twice a month and would contain culturally relevant produce tailored to Caribbean and Hispanic populations.

Abbreviations: EBT, electronic benefit transfer; USD, United States dollar.

1 Incentive amount and duration would be the same as with the Fresh Connect EBT card.

#### PIP analysis

Members of the research team (RP-E, SS-P, ATU) conducted the PIP analysis resulting from the codesign phase by articulating the specific program objectives and specific outcomes that were expected to be impacted, then systematically adding the activities and processes that would need to be in place for the program to meet its objectives, following causal thinking. The specific program activities outlined were *1*) community outreach; *2*) eligibility screening; *3*) enrollment and attendance in Supplemental Nutrition Assistance Program (SNAP) nutrition education (SNAP-Ed); *4*) provision of incentives; and *5*) redemption of incentives.

The nuts and bolts of each activity and the processes interconnecting them were fine-tuned over time by feedback gathered in the codesign LSs with support from the FED (Fidelity, Equity, and Dignity) principle workbook [33]. The FED principle posits the need for food prescription programs policies and programs to be designed with fidelity to the needs and wants of communities, with the principles of equity and dignity in mind.

#### Mapping of F4M PIP to the COM-B framework

F4M was expected to positively impact the primary outcomes, i.e., increasing produce purchase and intake with the aspirational goal of improving maternal-child health in partnership with other initiatives. The F4M PIP was mapped by the authors (RP-E, SS-P) to the COM-B framework to explain how the program was codesigned to achieve its objectives. The initial mapping was modified with iterative input from the other coauthors until a final consensus was reached.

### Pilot phase

#### Feasibility study

F4M was implemented following the PIP and tested for feasibility in the pilot study described below.

##### Selection criteria and recruitment

Following the F4M codesign phase, a pilot study was conducted to test the feasibility of F4M. Participants were included if they were *1*) Hispanic pregnant women in their first or second trimester of pregnancy; *2*) living in the greater Hartford area; *3*) aged ≥18 y; and *4*) receiving or eligible to enroll in WIC, SNAP, or Medicaid. Recruitment strategies included distribution of flyers and direct contact with HHC maternal and child programs, as well as other community-based organizations providing maternal child programs. Outreach recruitment efforts also extended to WIC offices and community events held in the City of Hartford and the Greater Hartford area.

##### Study enrollment

Participants were screened for eligibility and, if eligible, consented by phone. Once they consented, they were sent a link through text or e-mail to complete a 20-min online baseline survey in their language of preference (Spanish or English) and invited to participate in a nutrition education session. Participants were fully enrolled to receive their PRx benefits once they were consented, completed the baseline survey, attended the first nutrition education session, and chose a PRx redemption option.

##### Human subjects considerations

The procedures followed were in accordance with the ethical standards of the institution on human subjects research, and approval was obtained from the relevant committee.

The codesign phase of the study was deemed exempt from institutional review board (IRB) approval by Yale University on 17 January, 2022 (IRB protocol ID: 2000031956), and our partnering organizations agreed upon with this determination. Recruitment for the codesign phase started Thursday, 26 January, 2023, and ended Thursday, 20 April, 2023. Although it was not required (as the codesign study was deemed exempt by Yale’s IRB), participants engaged with the codesign phase consented in writing prior to the start of the LSs. The consent was documented and witnessed by a research staff member.

The pilot phase received IRB approval on 10 May, 2023, from Yale University (IRB protocol ID: 2000034840) prior to the start of recruitment. Consent for the pilot phase was obtained verbally. All the information was documented in a tracking spreadsheet, and participants were given a copy of the consent form (either physical or digital) after the consent process. The study recruiter and the participant were present together throughout the consent process, and the recruiter documented it.

Both community partners, the HHC and Wholesome Wave, requested for Yale to serve as the IRB on record as they did not have their own IRBs in place. Both partnering organizations helped prepare and reviewed in advance all materials submitted to the Yale IRB.

##### Nutrition education sessions

A series of 4 nutrition education sessions were offered to participants. Participants needed to complete the first session to be fully enrolled in the program; the remaining 3 sessions were optional. The HHC SNAP-Ed program developed the lessons, which were consistent with the 2020 Dietary Guidelines for Americans and lasted an average of 90 minutes. The topics included *1*) Healthy Eating for a Healthy Pregnancy; *2*) Making Healthy Food Choices using the Nutrition Facts Label, Food Safety, and Nutrition Tips; *3*) Dietary Strategies for Prevention and Management of Pregnancy Complications (e.g., gestational diabetes, hypertension); and *4*) Nutrition for Post-Partum Women and Infant Feeding. After each session, program staff conducted a cooking demonstration and food tasting of a healthy recipe. Pre- and posttests were applied, with the questions following a Knowledge, Attitude, and Practice modified format in which self-efficacy was used instead of practice as the final outcome.

##### Baseline survey

A baseline survey, originally designed to assess other PRx programs evaluated by our study team in California, was adapted to include a food frequency questionnaire as well as pregnancy-related questions. The baseline survey assessed household characteristics, barriers to fruit and vegetable consumption, level of difficulty when incorporating fresh fruits and vegetables into participants’ diet, fruit and vegetable intake, food insecurity, self-reported health status, and dietary patterns. Subsequently, the survey was carefully reviewed in English and Spanish and iteratively discussed by the coauthors. All survey translations were completed and reviewed by native Spanish speakers of the F4M team (ATU, RP-E).

Following these refinements, the survey was pretested for clarity and content by F4M team members (KG, AH-F, RP-E, SS-P, AH, KOD, ATU). Each team member received the survey via e-mail, and their recommended adjustments were discussed in a consensus meeting. Once consensus was reached and revisions made, the baseline survey was sent to pilot study participants (*n* = 20) in their preferred language (English or Spanish) through an online text or e-mail link, according to participants’ preference. On average, the survey took participants 30 min to complete.

##### Pilot study LSs

Approximately 10 wk into the 10-mo program, 2 LSs (*n* = 9 participants total) were conducted to understand participants’ experiences with F4M, with 7 participants attending the first and 2 attending the second session.

The senior author of this article (RP-E) moderated the LS, and the first author (SS-P) took notes throughout the sessions. To guide the discussion, the LS questions were listed on flip chart paper pasted to the wall in a room at the HHC where the session took place (Supplementary Material - Table 2). The first session lasted 73 min, and the second 38 min.

##### Participant satisfaction survey

A participant satisfaction survey, including perceived respect, was developed by the F4Ms team (KG, AH-F, RP-E, SS-P, AH, KOD, KG, ATU, ECR), drawing from the literature [11] and their in-depth understanding of the local context. The survey also collected data on participants’ opinions on the utilization of incentives and what they thought about the possibility of switching redemption options after trying the initial choice for some time. Similar questions from the baseline survey addressing fruit and vegetable consumption, barriers, and food insecurity were also included.

The development of the satisfaction survey followed a similar process as the baseline survey, including procedures for translations and pretesting. This 30-min survey was distributed via texting a Qualtrics online survey link to respondents’ cell phones ∼3.5 mo into the 10-mo program; 14 out of the 19 pilot participants completed the survey. Each week for 2 wk, reminder texts were sent to respondents who had not completed the survey. Respondents who had not completed the satisfaction survey by the third week received a phone call as a final reminder.

### Data analysis

#### Codesign and pilot study LSs

The qualitative data from the codesign and pilot study LSs were analyzed separately but using the same thematic analysis methodology. Analyses were carried out by coauthors representing the community-based organization and academic partners (AH, ATU, SS-P, and RP-E). To develop codes, we first used deductive strategies (e.g., utilizing predefined topics derived from the LS guide) followed by inductive strategies (e.g., reading and rereading the textual data and recoding to identify additional topics).

Following the development, refinement, and finalization of the codebook for the codesign phase, 2 members of the analysis team (AH, ATU) independently coded each transcript using the comment function in Microsoft Word. Throughout the coding process, the full analysis team (AH, ATU, SS-P, and RP-E) met to compare and discuss the coded segments for all transcripts as well as resolve any discrepancies to ensure consensus and consistency in the application of codes. When coding of all transcripts from the 3 codesign LSs was complete, the transcripts were uploaded to Dedoose, a qualitative analysis software. The transcripts were then coded in Dedoose to match the finalized coding in Word. Once the codebook was developed for the pilot LSs, the team opted to use Word only for reviewing coded segments, given the small number of transcripts. Two members of the analysis team (AH, ATU) created detailed narrative descriptions of the findings for each code and refined these descriptions with input from the members of the analysis team who moderated the LSs (SS-P, RP-E) to ensure that interpretations were consistent and reflective of the data. Finally, the descriptions were reviewed to identify and organize the findings into themes by the analysis team following a highly iterative approach.

#### Baseline and participant satisfaction survey

Both the baseline and satisfaction survey data were downloaded from Qualtrics and exported to SPSS to generate the variables’ frequencies.

## Results

### Codesign LSs

#### Description of participants

Table 2 shows that the participants attending the codesign LSs were Hispanic pregnant or postpartum women who were aged 32 y on average. Overall, 57% of the participants spoke only Spanish, 33% were bilingual, and 10% spoke only English. More specifically, in the first and third LS, most participants spoke only Spanish, whereas in the second LS, 22% did so. The proportion of single women ranged from 25% to 44% across sessions, and most had a high school education or more. The proportion participating in WIC ranged from 50% to 100% and in SNAP from 0% to 67%. The women came from 4 different countries or territories in Latin America and the Caribbean. Hence, the goal of including and receiving the input from a diverse group of Hispanic women was achieved.

**TABLE 2:** Sociodemographic characteristics of Hispanic mothers in Hartford, CT participating in FOOD4MOMS codesign listening sessions.

	LS1 *n* = 8	LS2 *n* = 9	LS3 *n* = 4
Hispanic	100%	100%	100%
Age (mean), y	31.6	32.2	
Pregnant	50%	56%	50%
Children aged <2 y	50%	44%	50%
Language spoken			
Only Spanish	87.50%	22%	75%
Only English	12.50%	22%	0%
English/Spanish	0	56%	25%
Unemployed	100%	67%	75%
Marital status			
Single no partner	25%	44%	25%
Married/living with partner	75%	56%	75%
Level of education			
High school or more	75%	100%	100%
Less than high school	25%	0%	0%
Currently working	0%	33%	25%
WIC Participant (yes)	100%	67%	50%
SNAP Participant (yes)	37.50%	67%	0%

Data shown as percentages unless otherwise indicated.

Abbreviations: LS, listening session; SNAP, Supplemental Nutrition Assistance Program; WIC, Special Supplemental Nutrition Program for Women, Infants and Children.

### Codesign qualitative findings

At the start of each LS, the moderator inquired about participants’ views on the importance of consuming fresh produce during pregnancy and whether a program like F4M would be of interest to their community. The key themes identified are presented below.

#### Importance of consuming fresh produce

Participants emphasized the vital role of consuming fruits and vegetables in providing essential nutrients for their infants during pregnancy and while breastfeeding. Underscoring the importance of healthy eating, they described how their own eating habits can profoundly influence their children’s dietary preferences. For example, 1 participant mentioned how much she liked fruits and vegetables, noting that her children also developed a strong preference for them.

“I am a fan of vegetables and fruits. And, since my children were born, after a year, I realized that they also loved vegetables and fruit... I see that what the mother eats before and after and during pregnancy does affect the child in one way or another.” (Codesign LS 1)

Furthermore, some participants stressed the importance of a healthy diet in preventing pregnancy-related complications or conditions. For instance, a participant shared her experience of being diagnosed with gestational diabetes, which prompted a shift in her eating habits.

“I had a pregnancy at [advanced age], then when later I was detected (with) gestational diabetes, and they [doctors] told me that it was due to bad nutrition, that I had to nourish myself better. In fact, they told me that the condition of how my baby was going to be born depended on that... if I have a bad diet of eating hamburgers, bread, things that are not good for my health.” (Codesign LS 3)

Finally, participants also expressed a keen interest in fruits and vegetables due to their natural and wholesome qualities, emphasizing the positive impact on overall health. They also viewed nutrition as important not only for their infants but also their families:

“I’m here because nutrition is important to me, both for this baby that is going to be born as well as for my daughter and for everyone in my family.” (Codesign LS 1)

#### Importance of having F4M

Upon hearing the overview of the program, participants’ collective response showed genuine appreciation for the program, recognizing it as a significant initiative, particularly for those facing financial constraints. Participants also commended on its potential to provide nutrition education tailored to pregnant women.

“I think it is very good. Well, I think it is an initiative to begin to nourish ourselves much better if we did not do it before, because now we are going to benefit ourselves and our baby.” (Codesign LS 3)

The inclusion of nutrition education sessions within the program was viewed very favorably by participants. They considered the sessions as ideal for addressing questions related to pregnancy and infant nutrition, as well as an opportunity to receive guidance on fruits and vegetables as an alternative to other foods, especially beneficial for individuals dealing with anemia, meat aversions, dietary restrictions, or other health conditions. Overall, participants stressed the need to make the program accessible to all pregnant women in a community setting, regardless of their socioeconomic or racial/ethnic background.

#### Experiences with similar programs

Participants shared their experiences with other food assistance programs such as WIC and SNAP, which support pregnant and postpartum women and their infants. Although participants generally expressed appreciation for these programs, many also identified areas in which WIC and SNAP could be improved. All participants enrolled in WIC valued the WIC EBT cards, but some voiced concerns about the limitations on eligible food items, especially when they included unhealthy options like sugary juices.

“In WIC, foods are restricted, even for fruits and vegetables, doesn’t it, it will be better if they allow a higher freedom to choose... For instance, I don’t agree that as part of the list of foods they give an immense number of juices, that are very high in sugar, which is not healthy...” (Codesign LS 1)

Participants hoped SNAP offices could provide information to pregnant women about places where they could purchase their cultural foods and refer them to other programs supporting pregnant women.

“At SNAP offices, they should be able to help you not only with application for SNAP, but if they see that you are pregnant, they should recommend you (of) places to go, such as Hispanic supermarkets....at these Hispanic places…” (Codesign LS 1)

Participants noted that not everyone enrolled in WIC also receives SNAP benefits, and not every pregnant woman in need meets the criteria for WIC, which they considered problematic. They emphasized how both WIC and SNAP benefits often fell short of lasting for the whole month, especially to purchase produce.

“Not everybody has a food stamp (SNAP), but most people have a WIC. It’s not enough money for veggie and fruit. You can spend it all for one week, and the other two weeks you’re running out.” (Codesign LS 2)

#### Locations for recruitment

Several potential program recruitment sites or channels were suggested, including WIC and SNAP offices across the greater Hartford area, obstetrics and gynecology clinics, community-based organizations, Hispanic supermarkets, local television channels, and maternal and child programs at HHC.

#### Incentive options

Incentives are a key aspect of a PRx. In the LS, we gathered participant feedback on 4 redemption options (EBT card, vouchers, online ordering, and delivery box) and whether the incentive amounts were deemed satisfactory.

##### EBT card

In general, participants strongly favored the EBT card over the other redemption options, primarily because the EBT card offers them the flexibility and autonomy to choose their preferred fruits and vegetables at grocery stores without any restrictions. Additionally, they anticipated that the EBT card would be convenient and easy for them to use, given their prior experience with the WIC EBT card system.

“[with the EBT card] I can go to the mall, to Walmart, Stop & Shop, and I can choose my own fruits and vegetables. This way, I can plan my weekly shopping based on what I am consuming, what I am buying, and what I need.” (Codesign LS 3)

##### Voucher

The preference for vouchers was comparable to the EBT cards, with participants emphasizing the importance of having easy to access stores that were close by accepting vouchers.

##### Online ordering

When asked, participants considered ordering produce online from a supermarket as a helpful option, particularly in case of emergencies, when they lacked transportation, or were physically unable to leave their homes due to recent childbirth or having children at home.

“In any case, [online order] could also be an option for me. [If] I wouldn’t be able to go to the store, some emergency maybe [I] wouldn’t [feel] good or something…I would try ordering it online.” (Codesign LS 3)

However, compared to using cards and vouchers, online ordering had a lower appeal to participants due to concerns about the quality of fruits and vegetables that supermarkets might choose for delivery and the presence of delivery fees.

Additionally, participants discussed delivery Apps such as Instacart. Some found it highly convenient, as it allowed them to stay at home and tend to their children while having groceries delivered to their doorstep. However, others raised concerns about potential challenges, especially for mothers who had low digital literacy and lacked access to phones, the internet, or technology support.

“I think it’s a good idea, but not the best, right? Because not everybody has access to Instacart or has access to the internet or phone. It’s like Uber. Everyone knows about Uber, but not everybody uses Uber, or not everybody knows how to place orders.” (Codesign LS 3)

##### Produce box

Some participants viewed the produce box as an opportunity for other members of their community.

“Well thinking about other people, it would benefit them if there were some distance [option], because if they can’t get there that day or something...” (Codesign LS 3)

However, they identified potential challenges with this option. On the one hand they were concerned about produce quality, spoilage, and waste when the box was received at a time when it was not needed. On the other hand, they also thought this option could prevent them from being able to get their produce when they needed it.

“But what if one week, you just want to make a specific dinner…I don’t have the ingredients I need now.” (Codesign LS 2)

When comparing the 2 remote incentive options, online ordering compared with produce boxes, many participants preferred online ordering as it would allow them to choose their preferred produce.

##### Incentive amount and duration

Participants believed that the proposed incentive amount of $100 per participant each month for 10 mo would be helpful in enabling them to purchase more produce. However, they also indicated that a higher amount would be even more beneficial, especially considering rising food prices and the fact that many families rely on a single income, making their food budgets tighter.

“It is a very good amount for any family, as a help, well I don’t know if it is enough, but for me it is good because the way things are now, all the vegetables [prices] have gone up too much and there are some families, some mothers don’t work, only the father works; so, the money is not enough but it [the incentive] is a great help…” (Codesign LS 1)

Furthermore, participants expressed a preference for extending the incentive program’s duration, which is currently 10 mo, to potentially up to a year. They noted that as their infants grow, they will require nutrition from complementary foods, making it a crucial period to ensure adequate consumption of healthy produce. Additionally, they reported that mothers experience many changes in their bodies after birth and require healthy nutrition during this period.

“I think that [incentive duration should be] up to the first year [postpartum]. It would be something reasonable that they [babies] already start eating…I consider that it would be important because…the baby’s nutrition continues, and the mother [body] wears out…” (Codesign LS 1)

Interestingly, participants would like for the incentive to be allowed to be used for the purchase of other healthy nutritious foods.

“I think that if they could extend it to buying other things like meats…my doctor wants me to eat protein so much.” (Codesign LS 1)

### Store options

Participants suggested several food outlets as potential redemption sites and shared specific reasons both in favor of and against each option (Supplementary Material - Table 3). Arguments in support of specific supermarkets or food retail outlets included lower prices, the quality and quantity of the produce selection, the availability of organic options, the eligibility for redemption of organic options through other benefits, and the convenience of the outlet’s location.

“Well, in my case, I already have WIC for my little one, so it makes it more feasible for me to do my shopping at Walmart or Stop & Shop. I can also choose what I need and keep track of what is missing.” (Codesign LS 3)

Conversely, reasons for opposing certain food stores included factors such as their seasonal nature, high prices, inconvenient hours of operations and locations, disqualification of organic options for redemption through other benefits, limited produce selection, and sanitation concerns.

### Nutrition education sessions

#### Importance

Participants strongly endorsed having nutrition classes and attending the first one as a requirement to receiving the incentive from the study. Providing information about nutrition while offering financial incentives can significantly help individuals make healthier diet choices in the context of their communities.

“It's very good [to have nutrition classes] because…how we live in the United States, people rushed all the time, and they think that what they are grabbing from the counter is the healthiest thing, and if they don’t know what is a protein, what is a vegetable, what is a cereal, if we can give them that information before we give them the money it’s going to be much more helpful.” (Codesign LS 2)

Participants also believed that having nutrition classes would significantly contribute to mitigating risks of poor pregnancy outcomes, diabetes, and obesity, which are pressing health concerns in their community.

“I like to be a little bit more aware of the nutrition part because it is important both during pregnancy and after pregnancy… because we are here with a serious obesity problem, which is not only during pregnancy but now [even for] children….” (Codesign LS 1)

The nutrition classes were seen as vital not only during pregnancy but also postpartum to benefit both the mother and the infant.

“We would also have to educate ourselves, according to how we are going to nourish ourselves or how we are also going to nourish the baby.” (Codesign LS 1)

#### Content

Overall, participants viewed having nutrition education as a crucial component for the program and maintaining health during and after pregnancy. Participants discussed various topics that nutrition education sessions could cover, including exploring new recipes, addressing pregnancy-related discomforts such as iron deficiency and nausea, and understanding the role of micronutrients. Participants were interested in learning and trying new recipes and cooking methods through an interactive and practical nutrition class.

“You could actually…show them one meal per day what would work for their nutrition in that one day. You know…simple stuff.” (Codesign LS 2)

Furthermore, participants were interested in learning about nutrient absorption, especially for critical nutrients such as iron and vitamins. A participant expressed her personal struggle with iron deficiency, emphasizing the importance of practical advice on dietary choices to maintain adequate iron levels during pregnancy.

“I was suffering from low iron, and I am still suffering from it; so, if I sit down and they (the nutrition class) give me the orientation, look, this is what you can eat…or they give your ideas of things that can help you to have good iron before delivery…it is very essential that they offer it.” (Codesign LS 1)

Some participants recommend to including ways to address challenges, such as persistent nausea and low iron levels during pregnancy, through healthy diets.

“I know that usually, they say that in the first trimester these discomforts should pass; I’m six months and I’m still the same, with the same discomforts… there should be very good information respect to that.” (Codesign LS 1)

#### Format

Participants explored various formats for conducting the nutrition education sessions. Most participants preferred the option of group classes, emphasizing the significance of having diverse viewpoints and the collective sharing of knowledge inherent in this format.

“You get better feedback with a group because of the fact that we have different ages, we got different opinions, we got different methods of doing certain things, and it makes it easier because we got more knowledge in the process.” (Codesign LS 2)

Some participants expressed a preference for in-person sessions, emphasizing benefits such as face-to-face interactions and real-time discussions. Remote sessions were preferred by individuals facing challenges that may restrict their ability to leave their homes, such as an imminent childbirth.

“I’m almost due to give birth and then I don’t know if I could leave my house…I have to get on the bus, wait for it and now if it’s cold; for me that would be it.” (Codesign LS 1)

#### Nutrition education through text messages

Participants expressed their familiarity with receiving text messages, particularly through WhatsApp, in other programs they had participated in. Participants agreed that text reminders would be valuable for efficiently tracking their EBT balances.

“That [balance reminder] is more important, everybody wants to read that. Like, ‘How much do I got left?’” (Codesign LS 2)

They also saw the potential of receiving recipes via text to experiment with different fruits, vegetables, and other ingredients not typically included in their meals. Nevertheless, some participants mentioned that they might unintentionally overlook nutrition tips and other messages, either by forgetting to read them or accumulating a backlog of unread content.

“[I am] so busy, or sometimes too tired to look at it, and it’s like... then when I get to it, it’s like a month later, or a week later, and I’m falling behind.” (Codesign LS 2)

**FIGURE 1:**
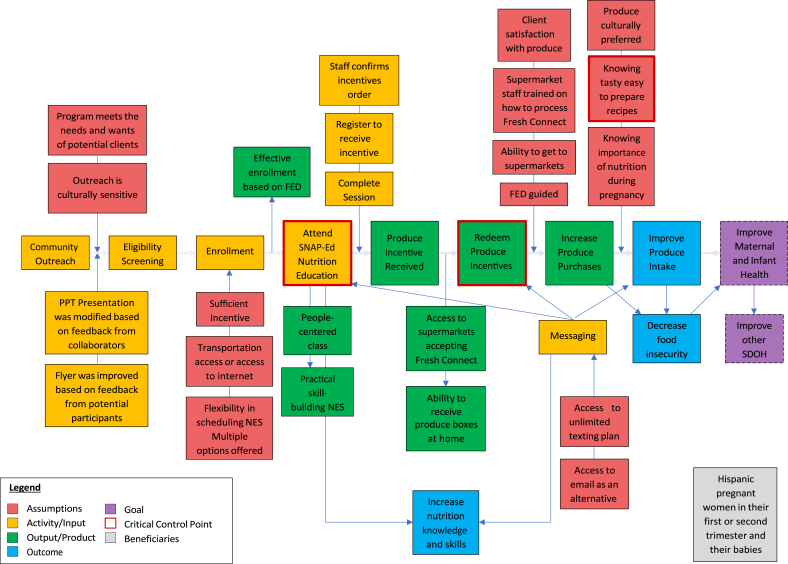
FOOD4MOMS Program Impact Pathway. Codesigned and tested for feasibility with low-income Hispanic women from Hartford, CT in their first or second trimester of pregnancy. FED, Fidelity, Equity, and Dignity; NES, nutrition education session; PPT, Powerpoint; SNAP-Ed, Supplemental Nutrition Assistance Program for Women – Education.

The PIP for the F4M resulting from the codesign phase illustrates each step of the program and corresponding critical quality control points (Figure 1). The first key step is community outreach to socialize the program followed by screening and enrollment of participants who then have to attend a mandatory nutrition education session to be signed up to receive their EBT card or receive their produce at home. Once their EBT card is activated o they receive produce at home, they can then get the produce, prepare it, and consume it to improve overall produce intake. The 2 critical quality control points identified were participants’ attending the mandatory nutrition education session and redeeming their produce benefits. Furthermore, the diagram indicates the conditions that need to be met for the quality control points to allow the participants’ journey in the program to flow smoothly. The mapping of the PIP to the COM-B framework strongly suggests that the F4M program, if implemented with fidelity, would be able achieve its objectives to improve produce purchases and intake. The components that mapped to the Capability dimension were the easiness of redemption of the PRx in the form of a debit-like card and the skills building on how to prepare fruits and vegetables through the nutrition education sessions. The Opportunity was provided by being able to redeem the PRx in supermarkets familiar to them or having the choice to have the produce delivered at the participants’ doorsteps and offering the nutrition education session through a trusted community-based organization. We posited that the motivation to engage with the program was driven by the Capacity and Opportunity factors combined with the perception of benefit to the infant and mother and the $100 per month offered for10 mo to purchase produce. The PIP is systematically presented in the following pilot study section.

### Pilot phase: feasibility study

#### Community outreach

HHC conducted successful outreach program recruitment activities among community partners—WIC program, a local hospital, and community-based organizations—offering services to low-income pregnant women. As suggested by one of the partners, a QR code was included in the study’s recruitment flyer to access a referral link through a Microsoft Form. Recruitment was also conducted at HHC through its maternal and child programs. In addition, program staff distributed bilingual (English and Spanish) flyers in strategic locations such as the HHC front desk, HHC outreach events, and WIC offices.

#### Eligibility screening and enrollment

**FIGURE 2:**
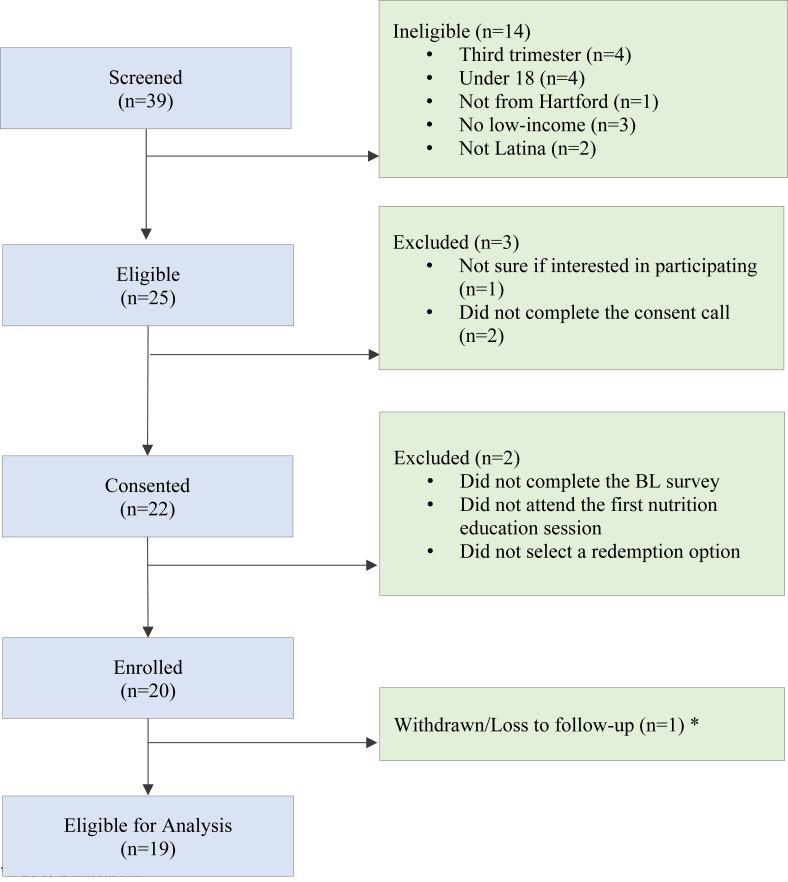
FOOD4MOMS enrollment flow chart. Pilot study among Hispanic pregnant women in Hartford, CT. ∗Note: One participant had to be withdrawn from the study. BL, baseline.

Participants underwent a screening process to determine eligibility for inclusion based on *1*) being Latina/Hispanic; *2*) in the first or second trimester of pregnancy; *3*) aged ≥18 y; *4*) living in the Hartford area and having a low income as proxied by participating or being enrolled in WIC, SNAP, or Medicaid; and *5*) speaking English or Spanish. The majority of participants were recruited from WIC and HHC Maternal Health Programs. There were 39 screenings, with 14 potential participants deemed ineligible. Ineligibility reasons included being in the third trimester of pregnancy, aged <18 y, not residing in the Hartford area, not meeting low-income criteria, and not identifying as Hispanic. Among the remaining 25 eligible participants, 3 were excluded due to lack of interest or failure to respond to the consent call. Of the 22 who consented, 20 were enrolled, and 19 remained throughout the pilot study (Figure 2). The reasons for excluding 2 of the initial 22 participants who consented was that they did not fulfill enrollment requirements, such as completing the baseline survey, attending the first nutrition education session, and selecting a redemption option. Among the 20 enrolled participants, 1 individual dropped from the study the study due to a miscarriage. This participant was informed that she would remain eligible to activate and use her Fresh Connect EBT card and that she could continue using her incentives for 4 mo.

#### Incentives

Based on the extensive feedback from the participants across LSs, and what was logistically feasible to do by the program, a decision was made to offer participants a choice between the EBT card or home-delivered produce boxes with both being equal in value. Specifically, participants were presented with a choice between 2 redemption options for their incentives: the Fresh Connect EBT card and the Umoja Produce Box. The Fresh Connect EBT card was sent by mail to their homes, along with instructions for activation. Upon activation, an initial $100 was loaded onto the card, followed by an automatic monthly addition of $100 at the start of each month. Participants received notifications via text from Fresh Connect when the card was reloaded. Any unused funds at the end of each month did not carry over and were forfeited. The Fresh Connect EBT card was exclusively designated for the purchase of fresh fruits and vegetables and was valid only at Walmart and Stop & Shop, the only stores with which Fresh Connect had contracts in Connecticut.

Selecting the Umoja Produce Box meant that the produce boxes would be delivered to their homes twice a month and that the cost of the produce provided to them would also be $100 per month. Participants could choose from 3 box options: more vegetables, more fruits, or an even mix of fruits and vegetables. These boxes were tailored by the study team to provide culturally relevant produce tailored to the preferences of Hispanic people. Interestingly, in the pilot study, all participants opted for the Fresh Connect EBT card. Participants were provided a handout with information and contact phone numbers to report lost cards or problems with activating their card to F4M staff.

#### Nutrition education session attendance

**FIGURE 3:**
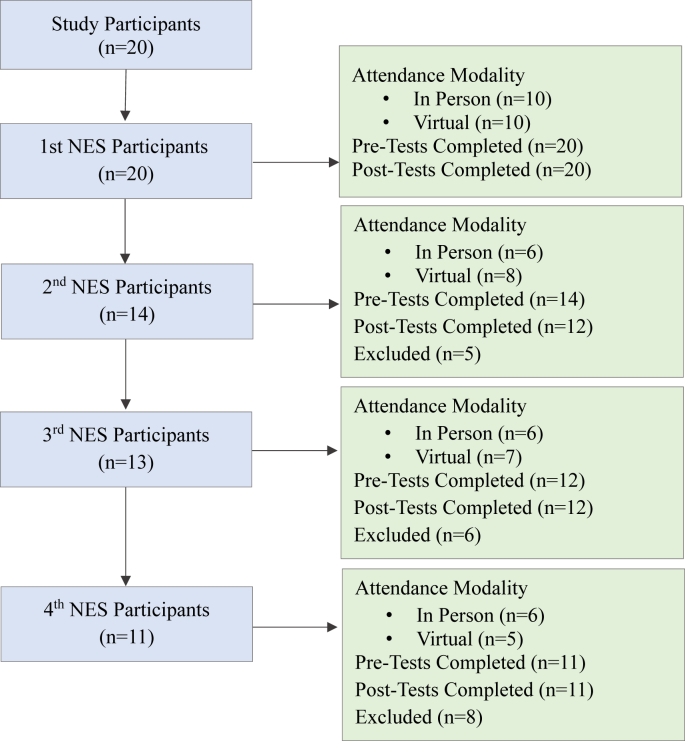
FOOD4MOMS participants’ attendance by nutrition education session and mode of participation. Participants were Hispanic pregnant women in Hartford, CT. NES, nutrition education session;

All 20 recruited participants attended the first nutrition education session, and between 11 and 14 participants attended the subsequent optional sessions (Figure 3). For each session, approximately half of participants attended the session in-person and the remaining half virtually. Almost three-quarters (74%) attended the second, 68% the third, and 58% the fourth optional sessions. The overall attendance rate for all nutrition education sessions was 75%.

#### Participant redemption rates

In December 2023, all pilot participants had successfully received their cards, and every participant in the pilot program has redeemed their incentive at least once.

#### Text messages

Participants received weekly text messages containing nutrition tips, recommendations, and recipes as supplementary educational resources. Text messages were also used to notify them when their cards had been reloaded and to remind them toward the end of each month to utilize all their incentives.

### Pilot participants’ assessment of program

#### Pilot study LS

##### Motivation: reasons for joining F4M

Participants were interested in joining F4M mainly because of the nutrition education coupled with the financial support that F4M provided. Participants recognized the significance of the nutrition education sessions in maintaining their and their infant's health during and after pregnancy. They highlighted the importance of understanding how to address pregnancy-related issues such as appetite loss and nausea, noting that increased access to healthy foods, particularly fruits and vegetables, could help mitigate these concerns.

Additionally, participants were motivated to join the program for financial reasons, given their perceived high cost of nutritious foods, especially fruits and vegetables. They believed this assistance would enable them to provide better nutrition for their infants. Furthermore, they anticipated that the incentives and recipes provided could enhance the consumption of fresh fruits and vegetables.

“What caught our attention the first thing is that they helped us – well, especially the $100. That for me was, it’s very much an experience. In other words, because apart from the fact that they give you – economically, they teach you how to eat healthily, not only for you but for your baby.” (Pilot LS 1)

##### Positive staff interactions

Participants appreciated the clear and timely presentation of the study, particularly highlighting the thoroughness of the recruitment and consent calls conducted by HHC staff, as a participant stated, “I liked it because from the beginning they were clear in detail about what the program was going to be like.” (Pilot LS 1)

Overall, participants felt the program was well-organized, offering comprehensive guidance throughout different phases, including recruitment, consent, baseline survey, nutrition education sessions, card usage, and activation. They commended the staff's detailed and clear explanations of the study components, ensuring them that their comfort was our top priority and that they had the option to withdraw at any time. The communication received via multiple channels—calls, emails, and text messages—were appreciated, especially the text messages providing them with benefits balance reminders and recipes for preparing fruit and vegetable dishes. The participants appreciated the study consent calls, which allowed them to comprehend all study details before deciding to participate.

“The program has been very organized. From the beginning, there was much guidance in everything that would happen. Consecutively, the cards arrived safely, as did the balance.” (Pilot LS1)

They also valued the reminders for the nutrition education sessions and praised the HHC staff for their kindness. Participants found the baseline survey to be appropriate in content and duration, reporting that questions were clear and easy-to-answer and that that did not cause discomfort. Participants were pleased that it prompted self-reflection on their diet, especially when realizing their low intake of fruit and vegetables.

“I liked the survey because it makes you think about what you are eating, what you should eat, and if you are eating well…I like it because it makes you reflect…” (Pilot LS 1)

Furthermore, they appreciated the nutrition class presenters for their skills in explaining materials and providing insightful guidance in response to participants’ questions.

#### Easy-to-use incentives

Participants expressed a clear preference for the EBT card, highlighting that it afforded them the autonomy to “decide when to shop for vegetables and fruits,” “see what I choose,” and “buy whatever I wanted between fruits and vegetables.” (Pilot LS 1)

Although participants encountered some technical difficulties with the WIC EBT card, they found the Fresh Connect EBT card from this study to be very user-friendly during the checkout process at Walmart and Stop & Shop, whether they opted for self-checkout or a cashier.

“I have only used it at Walmart and I have not had any problems. Everything is fine with the card at the time of payment, I go to the checkout where you pay it yourself. I haven’t had a single problem with the scan, everything is in order.” (Pilot LS 1)

Regarding their preference for grocery stores, most participants favored Stop & Shop. This preference was primarily due to the greater accessibility of local Stop & Shop stores for most participants and the perceived wider selection of fresh fruits and vegetables compared to Walmart stores.

#### Engaging learning experiences

Participants appreciated learning about their infant’s growth and brain development and being reminded about the importance of healthy eating during pregnancy and how to maintain it. They also valued the advice given on healthier meal options, emphasizing the importance of maintaining a varied and healthy diet.

“I liked [the nutrition education session]. I have learned a lot and I like that. And more about things – there are things that…even if we…have had children, we have learned a lot here in [these] classes, because in nutrition sometimes we get out of control. We eat…and…sometimes we don’t know what we are eating.” (Pilot LS 1)

Participants found the healthy recipes shared by the program to be helpful and emphasized the knowledge they acquired about nutrition, diverse meal preparations involving fruits and vegetables, and food safety. Consequently, they felt better informed and more cautious when making dietary choices, as well as more empowered to prepare new, healthy meals.

“Well, the classes caught my attention. I had honestly never been to a nutrition class. So, when they asked me, ‘Do you know about nutrition?,’ I said, ‘Yes,’ but in reality, having a class is more beneficial...For example, [for the soda consumption], I didn’t know how much sugar it had, and when she [research staff] shows it to you, and you ask, ‘Can I drink all that sugar? All that soda?’ And then, the classes become very interesting.” (Pilot LS 1)

Participants specifically mentioned that they learned how to read food labels and became more attentive to the ingredients. This has enabled them to make informed consumption decisions, particularly regarding sugar, salt, and saturated fat content listed on food labels.

“What I liked the most was seeing the label how much of what you buy, how much sugar it contains, calculating the sugar, because sometimes you…don’t look and don’t read. And here I have learned that you have to read what is in it, the proteins, the vitamins and everything.” (Pilot LS 1)

Regarding the format of nutrition education sessions, all participants expressed a preference for in-person over virtual sessions. They enjoyed the face-to-face interactions and the engaging environment during these sessions. Meanwhile, they found the remote option to be more complex and sometimes encountered technical difficulties. Moreover, participants mentioned that participating remotely during in-person activities could be challenging, as it was easy to overlook the presence of virtual participants whereas the in-person activities were happening.

“I liked that we had the options of doing it virtually or coming in person. I once did virtual, and I liked doing it more in person because you can see. Sometimes on the screen it was a little complicated, and being here [in person] they don’t leave you hanging, they give you the papers so you can follow what they are talking about, you take it home and you can see, ‘okay, this I can consume it, this is not what hurts.’... I liked that.” (Pilot LS 1)

#### Positive program impact: dietary habits and personal finance

Participants reported positive outcomes from the pilot program, stating that the F4M project not only helped them and their families consume more fresh produce but also alleviated their financial burden, particularly with the rising costs of produce. Participants indeed expressed deep gratitude for the study, stating that it allowed them to prioritize nourishing themselves and their infants more effectively.

“It [the program] has helped me a lot to consume more vegetables and fruits…I made this smoothie of juices, juices, fruit juices every day. Not just for me, also for my husband and my daughter. And I sent to his work, his banana juice, strawberry juice... And in the case of meals, it helped me a lot with fruits and vegetables.” (Pilot LS 1)

This increase in produce consumption was attributed not only to financial assistance but also to the nutrition education classes and recipes offered by the F4M. The nutrition education taught participants innovative ways to prepare fruits and vegetables and underscored the significance of including them in their diets.

“Because of the guidance they give, the recipes, also the incentive. The truth is that it has made me consume more [fresh fruits and vegetables] than I did before. Well, because sometimes not only for money, but also for how I prepare them, how I can ingest them. But now I’m really very grateful and so far so good.” (Pilot LS 1)

Furthermore, participants greatly appreciated the financial support provided by the incentive, especially with the increasing food prices. They emphasized that the incentives enabled and motivated them and their families to incorporate more fruits and vegetables into their diet and ensure access to fresh fruits and vegetables.

“[The incentive is] very good. You buy more fruits, consume more fruits and vegetables. And for the children above all. I am a single mother and I live alone with my children and well, I work for rent and bills and all that, and $100 is a great help for ten months.” (Pilot LS 1)

Interestingly, participants recognized a synergistic relationship between the EBT card provided by the WIC program and the F4M Fresh Connect card. They pointed out that these resources combined to offer them greater flexibility and a larger budget for purchasing a variety of foods, allowing them to strategically allocate funds toward fresh produce and other essential food items.

“In my case, on the weekends when I do my shopping, I go through the WIC products first, let’s say the milk, then the cereal, and then the fruits or vegetables. And I go through all those products and pay it first with WIC, and I see that I lacked balance for vegetables and fruits, and there I go through Fresh Connect.” (Pilot LS 1)

### Potential program improvements

Participants provided suggestions for program improvements including expanding the available stores for redemption beyond Walmart and Stop & Shop and displaying the remaining balance on receipts during checkout or via text messages. Additionally, they proposed extending the study duration beyond the initial 10 mo, suggesting an extension ≤1 y postpartum or until their infants reached the age of 3 to 4 y. Increasing the incentive amount was also suggested, along with allowing the rollover of remaining incentives for situations in which transportation or mobility issues hindered redemption. Finally, there was a suggestion to include a concise guide on downloading the Zoom application for the virtual nutrition education sessions.

### Client satisfaction survey results

**FIGURE 4:**
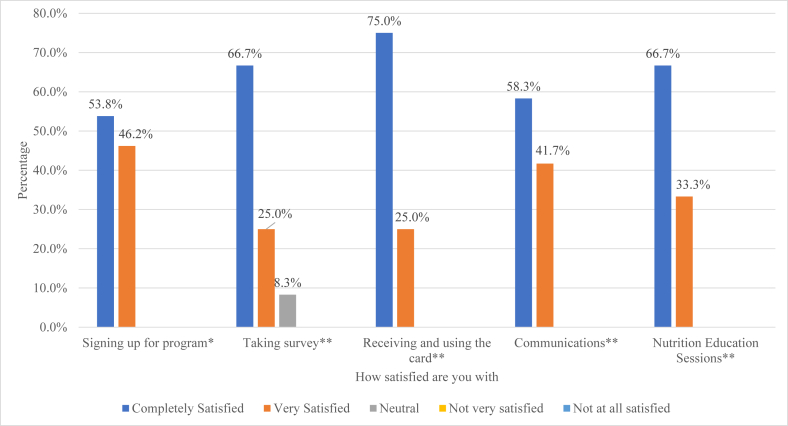
FOOD4MOMS Program client satisfaction among pregnant Hispanic women in Hartford, CT. Notes: ∗1 response missing; ∗∗2 responses missing. Sample size may not add up to total sample due to missing data. The percentage reported represents the valid percentage for the sample for each variable.

Of the 19 pilot participants, 14 responded to the client satisfaction survey, offering insights into their overall assessment of the F4M program, including its impact (Figure 4). All respondents reported that they were very satisfied with the F4M program, that they would strongly recommend it to other women in their community, and that the Fresh Connect card was very easy to use. They reported that the PRx has been very helpful at improving both their own and their family’s dietary habits (78.6% and 21.4%, respectively). Among respondents, 53% to 75% indicated they were completely satisfied with all program components, while 25% to 46% indicated they were very satisfied. All participants reported that they were *very* or *completely* satisfied with signing up for the program, receiving the card, communications, and the nutrition education received**.**

Participants were also asked whether they encountered any difficulties related to the program components. One respondent (7.2%) reported difficulties using the card, 2 with attending classes (16.7%), and 1 with taking the surveys (7.2%). When asked about the specific nature of the challenges faced, 1 respondent explained that she was unclear where she could use the card, 1 had difficulties logging onto Zoom and confronted scheduling conflicts with simultaneous appointments, and 1 mentioned she depended on public transportation to attend the nutrition classes. One mentioned that the survey was long and that she was prompted to start the survey again when she believed she was about to complete it.

Most respondents said checking the balance on the card (78.6%) and understanding how to benefit from the program (71.4%) was “very easy,” while approximately one-fourth of participants indicated they were “only somewhat easy” (14.3% and 21.4%, respectively). Two participants mentioned that it was “somewhat hard” to check balance on card (7.1%) and to understand how to benefit from the program (7.1%).

Over one-third (35.7%) of respondents considered the F4M incentive amount more than enough, 42.9% deemed it just enough, 14.3% felt the incentive was not enough, and 7.1% stated they did not know if it was enough. Participants reported that there was high-quality produce at the stores where their redeemed their PRx “all of the time” (14%), “most of the time” (57%), and “a few times” (29%). Furthermore, slightly less than half of the respondents reported there was sufficient variety and their preferred types most of the time (43%), while the remaining reported either “all of the time” (29%) or “a few times” (29%).

## Discussion

Our study demonstrated that community-engaged program codesign principles are essential prior to launching PRx programs. In our study, this approach likely explains why the piloting of the codesigned program confirmed its feasibility and strong client satisfaction.

Our innovative mixed-methods community-engaged approach centered around a close-knit community-academic partnership documented how a well thought out consultation process can help codesign a feasible and well-liked evidence-based PRx that meets the FED principle [23,33]. Hence, this work adds substantial knowledge to the field [[34], [35], [36], [37], [38], [39], [40], [41]].

The first phase of this study showed the steps that need to be taken to ensure PRx programs meet the needs and wants of communities through a community-engaged codesign process. The LS that informed by the PIP process allowed the partners to confirm directly from potential clients that a PRx for pregnant and postpartum Hispanic women was very much needed. It also allowed for the potential clients to express their desires on behalf of their community on the specific preferences for ways to receive the PRx incentive and nutrition education and useful information via text messages. Although a program codesign limitation was that it was not always feasible to provide participants’ top preferences, the LSs allowed for understanding if the next best options would still be appropriate from the perspectives of the clients. For example, whereas the top preference was to receive the incentives in the form of an EBT card that could be used in any supermarket or local food store, this option was not available in the marketplace.

A second popular option was receiving vouchers for their neighborhood supermarket, and this was not logistically possible either. Rather, they were offered an EBT card that could be used in a large supermarket chain and a large retail store selling food at diverse locations in Hartford. Participants understood the reasons and indicated that they agreed to that option but that they also thought that a home delivery option, such as online supermarket ordering or a produce box, may be helpful for other women in their community with severe transportation and time barriers. Although the produce box option was not universally popular, it was the only home delivery option that could be incorporated into the program. None of the women chose this option during the pilot phase; hence, the program team is currently exploring the feasibility of other home delivery alternatives that could be implemented via F4M. This key finding highlights the challenge of community-engaged codesign when there are strong logistical constraints. Findings from F4M can be used by PRx program funders and policy makers to help facilitate the removal of such constraints.

Importantly, the findings from the codesign LSs highlight the strong sense of solidarity among women, as participants specifically asked for F4M to be available to all pregnant women regardless of their national origin, documentation status, race, or ethnicity. Additionally, they clearly endorsed the PRx approach combined with nutrition education as they felt that it was crucial not only to provide access to more produce but also to nutrition education for women to understand how to prepare and consume a healthy diet during pregnancy and how it affected their health as well as the growth and development of their offspring. Based on the feedback obtained, a decision was made to deliver the nutrition education sessions following a group format and to offer the option of in-person or virtual participation, facilitating the exchange of ideas within a diverse group and ensuring accessibility to nutrition education for all participants.

Participants also felt that it was important for the PRx to be run by a community-based organization and not a hospital and medical facility, perhaps reflecting the different types of experiences they have had with services in both settings. The implication of this key finding is that it is important to consider reimbursing PRx services directly to community agencies that provide health services or that partner with a health care system.

Our study also demonstrated the importance of a small pilot feasibility study of the codesigned program to ensure that any further adjustments needed would be in place before testing the F4M PRx in a study with a larger population. The findings from our pilot study indicated that there was strong uptake of the program among clients because it offered financial and health benefits, was very user-friendly particularly with regard to receiving, activating, and using the EBT card, and was person-centered and respectful. Clients were also satisfied with the supermarkets that they could redeem their produce benefits, including their experiences with cashiers. As such, our findings indicate that programs that promote positive experiences and high satisfaction among clients may achieve greater client engagement. This is important given that a major challenge facing the Food is Medicine field is how to improve redemption rates of financial incentives among clients, which have been found to range from <20% to >80%, with most studies reporting redemption <50% [[34], [35], [36], [37], [38]].

Participants appreciated the nutrition classes during pregnancy, which is fully consistent with a recent study from Texas [39], and remembered key messages but did have some recommendations for improvement. The main changes recommended were to not mix in-person with virtual participants in the same nutrition education session because it was easy to forget that there were people that had joined virtually once the in-person activities, such as cooking demonstrations, had begun.

Regarding the PRx incentives, the participants also asked if it was possible to eliminate the requirement to spend their full incentive every month as several of them had already “lost” money because of having some of it left at the end of the month. Unfortunately, this recommendation could not be implemented due to the limitations of the Fresh Connect card program.

Overall, participants were highly satisfied with the program which is consistent with some [42,43] but not all [40,41] previous PRx studies. They also indicated that the F4M was not redundant with but rather that it strongly complemented WIC and that it was easier to benefit from. Hence F4M offers lessons that can be used by WIC to improve its clients’ experiences and impact [44,45].

In response to feedback from participants during the pilot LS, we are making balanced information more accessible. Specifically, we have incorporated the remaining balance details into the balance reminder texts. with a Python script that leverages EZ Texting API to quickly send batch text messages to participants.

In sum, this study demonstrated how community-engaged approaches can be used to effectively codesign and pilot test the feasibility and satisfaction of a PRx for pregnant Hispanic women. This methodology is being manualized into a toolkit so that it can be used to design PRx targeting different audiences in different settings. F4M has now entered its full evaluation phase to assess impacts on produce incentive redemption, purchasing, and consumption in the population of focus. A potential limitation of our study is that findings may not be extrapolated to other places where diverse Hispanic communities live across the country. However, the community-engaged methods used for program codesign and pilot feasibility testing can be applied everywhere.

Moving forward, it is important to continue identifying if and how PRx may improve food and nutrition security at a family level. Special attention needs to be made to understand if these programs also help reduce sugar-sweetened beverage and overall consumption of ultra-processed foods. It is also important to further research the optimal monthly allotment in terms of amount of money and whether it should be further restricted to the most nutrient-dense fruits and vegetables. Finally, future studies are needed to understand if and how the nutrition education sessions magnify the impact of the PRx benefit.

In conclusion, F4M was codesigned with strong input from the community and tailored to their needs. This approach likely explains the successful piloting of the feasibility of F4M and the strong satisfaction from clients participating in it.

## Author contributions

The authors’ responsibilities were as follows – RP-E, SS-P, KOD: designed the study; RP-E, SS-P: cowrote the first manuscript draft; ATU, AH, ECR, RP-E: analyzed the qualitative data; KG, AH-F: analyzed the quantitative data; and all authors: read and approved the final manuscript.

## Funding

This study was funded by Wholesome Wave with a grant received from the Unites States Department of Agriculture GusNIP initiative. KOD, ECR, and RP-E received partial support from the Yale-Griffin Prevention Research Center Cooperative Agreement Number 5 U48DP006380-02-00 funded by the United States Centers for Disease Control and Prevention, Prevention Research Center Program (PI: RP-E). Its contents are solely the responsibility of the authors and do not necessarily represent the official views of the United States Centers for Disease Control and Prevention or the United States Department of Health and Human Services.

## Conflict of interest

RP-E reports financial support was provided by Wholesome Wave. RP-E reports a relationship with Yale-Griffin CDC Prevention Research Center that includes funding grants. The other authors declare that they have no known competing financial interests or personal relationships that could have appeared to influence the work reported in this paper. Rafael Pérez-Escamilla is a Deputy Editor of Current Developments in Nutrition and played no role in the evaluation of this manuscript.
