# Supplementary material for: Community-Engaged Codesign and Piloting of the FOOD4MOMS Produce Prescription Program for Pregnant Latina Women
Source: Curr Dev Nutr. 2025 Feb 19;9(3):104572. doi: 10.1016/j.cdnut.2025.104572 (PMC11938078; doi:10.1016/j.cdnut.2025.104572)
Supplement: multimedia component 1 [file mmc1.docx]

Supplementary Material for article Community Engaged Co-design and Piloting of the FOOD4MOMS Produce Prescription program for Pregnant Latina Women by Segura-Pérez, S et al.

Supplementary material 1: Co-design listening session guide

| Topics | Example Questions Asked in Co-Design Listening Session (CD LS 1) (n=8) | Example Questions in CD LS 2 (n=9) | Example Questions in CD LS 3 (n=4) |
| --- | --- | --- | --- |
| Each listening session began with offering the participants a comprehensive overview of the proposed F4M program design, then delved into more detailed questions about various program steps. The LS 2 and 3 discussions built on the input received during the previous sessions. | | | |
| General Knowledge Assessment | *What do you think of this program?* | *What do you think about the produce prescription program?*  *Do you think fruits and vegetables are important for maternal nutrition during pregnancy?*  *Do you think moms would benefit from more money to get more fruit and veggie, or they're already getting enough from other programs?* | *What do you think of this program?*  *Do you think it's a program that is going to be of interest to your community?* |
| Previous Experiences with Similar Programs | *Do you know of any of these Hispanic Health Council programs?* | *Have you received cards like these WIC or SNAP?* | N/A |
| Recruitment Methods | *Where do you think Hispanic women could be encouraged to participate in this program?*  *Do you feel indifferent, or would you prefer to go to a hospital to receive this program?*  *Would women be recruited starting in the last trimester of pregnancy?* | *What do you think about recruiting the moms for this food prescription program through the Hispanic Health Council in Maternal and Child Health programs? Then what about the WIC clinic?*  *Are there other places that would be good to offer the program to women?* | N/A |
| Incentive Options | *What do you think of the incentive? What do you think of the 10-month duration? Do you think $100 a month is enough for the purpose of the program?*  *Do you think it should be for more fruits and vegetables? or could it be used for other types of food?*  *Have you received any of these cards or other programs from WIC? Do you think it's a good mechanism to give the incentive to moms or there are other ways to give it to moms?*  *Which supermarkets do you think would be the most suitable to buy the fruits and vegetables?*  *Have you had food delivered to your home? do you have to wait for hours to see what time they arrive?*  *Does the food box delivery sound like something you would be interested in?* | *What do you think about the inventive options?*  *For those of you who have had experience with Instacart, what has been your experience with the service?*  *What do you think about getting the boxes twice per month?*  *Which supermarkets would you think would be good options for fruits and vegetables, and which ones are not?* | Participants were asked to rank the produce redemption options that would be offered through F4M: one involving the direct purchase of produce at a supermarket or retail store (an EBT card or a paper voucher), and a second option involving produce delivery at home (online supermarket ordering or a produce box).  Once that ranking was completed a group discussion followed to gain additional insights on how this decision would affect the participants themselves and other women in their community. |
| Nutrition Education | *Do you think it is a good idea as part of this program to offer and expect the participants to come to the nutrition classes? Do you think a group class is a good idea?*  *What if it was done online, could you do it?*  *Do you think it's a good idea to send text messages [include nutrition knowledge] by cell phone?* | *What do you think about linking the delivery of incentives to participation in nutrition classes?*  *How do you envision the nutrition classes? Should they be conducted on a one-on-one basis or in a group setting? Do you think we should offer hybrid classes or only in-person sessions?*  *What types of group activities do you think would be engaging? How about cooking demonstrations or preparing healthy recipes together?*  *During your pregnancy, what questions did you have about nutrition, or what needs did you experience that you believe pregnant women should be informed about—things you wish you had known during your own pregnancy?*  *Do you think people would be receptive to receiving text messages that include nutritional information?* | *Tell me a little bit, for example, is nausea during pregnancy or gestational diabetes a topic that you are interested in learning more about? What topics would you be most interested in?*  *How do you envision these classes? Could they be one-on-one, in groups, interactive cooking activities, or sharing recipes? Do you think it should be in person or could they also be online? What ideas do you have?* |
| Wrapping-up | *Do you want to add anything else?* | *Anything else anyone would like to comment on?*  *Would you choose to enroll in a program like this?* | N/A |

Supplementary material 2: Pilot listening session guide

1. **Recruitment Experiences**

*How did you hear about this program?*

1. **Reasons of Joining F4M**

*Could you tell me what was the main reason you joined this program?*

1. **Experiences with Consent & Survey**
   1. **Consent Process**

*What did you think of the consent call for you to enter this project? Did they give you enough detail for you to understand?*

- 1. **Survey Experiences**

*What was your experience with the survey? It includes quite a few questions about you and your home, any barriers to accessing fresh fruits and vegetables, as well as questions about the topic of food safety and security. Were there any questions that made you uncomfortable?*

1. **Incentive Options**
   1. **Available Options**

*How were the options explained to you? How did you like the way they explained the Fresh Connect card? Was it clear or did you already know how it worked since you received it?*

- 1. **Incentive Preferences**

*Did any of you choose the houses option of having a box sent or did you all choose Fresh Connect? Did you like using the Fresh Connect card? Is there anything about Fresh Connect that you don't like very much, or that you would like to be different?*

1. **Nutrition Education Session**

*What has been your experience with the nutrition classes? Could you please tell me what you liked the most, what you liked the least about the nutrition classes? Have these classes kept your interest? What could we do to improve the virtual experience?*

1. **Text Messages**

*Do the text messages from the program help you?*

1. **Redemption Experiences**
   1. **Shopping Experiences**

*When you have gone with your card and go shopping, what has been your experience with the cashiers in general? What about the experience of going to buy your fruits and vegetables with Fresh Connect card? Have you ever had problems with self-scanning with the card?*

- 1. **Shopping Locations**

*If we compared the experience with Stop and Shop and Walmart, which is better?*

1. **Program Outcomes**

*We are also very interested in knowing whether this program has helped you and your family consume more fruits and vegetables. Or, if you already consume a lot, whether it still helps because you have more money to use for other things. What can you tell us about your consumption of fruits and vegetables?*

1. **Future Improvements**

*What would you recommend improving this program?*

Supplementary material 3: Store Options

Participants suggested several food outlets as potential redemption sites. Here is a comprehensive list of the proposed locations, accompanied by specific reasons both in favor of and against each option.

| **Store Options** | **Specified Reasons in Favor** | **Specified Reasons Against** |
| --- | --- | --- |
| Aldi’s | - Lower Prices - Quality of Food & Vegetable Selection |  |
| Big Y | - Organic Options Qualify |  |
| CTown | - Quality of Food & Vegetable Selection - Quantity of Food & Vegetable Selection - Location/Convenience |  |
| *El Mercadito* (Latino supermarket) | - Lower Prices |  |
| Farmers’ Markets | - Offers Organic Options - Quality of Food & Vegetable Selection | - Seasonal - Higher Prices - Hours of Operation - Location/Convenience |
| Key Foods | - Quality of Food & Vegetable Selection - Quantity of Food & Vegetable Selection - Location/Convenience |  |
| Price Rite | - Quality of Food & Vegetable Selection |  |
| Save A Lot |  | - Cleanliness |
| Shop Rite |  | - Location/Not Convenient |
| Stop & Shop | - Quality of Food & Vegetable Selection | - Higher Prices - Organic Options Don’t Qualify |
| Walmart | - Location/Convenience | - Quantity of Food & Vegetable Selection |
| Whole Foods | - Offers Organic Options |  |
